# Recalibration of hand position sense during unconscious active and passive movement

**DOI:** 10.1007/s00221-017-5137-7

**Published:** 2017-12-14

**Authors:** Zakaryah Abdulkarim, H. Henrik Ehrsson

**Affiliations:** Department of Neuroscience, Karolinska Insitutet, Retzius väg 8, 171 77 Solna, Sweden

**Keywords:** Multisensory, Perception, Sensory recalibration, Vision, Proprioception

## Abstract

Precise knowledge of one’s limbs’ position in space is fundamental for goal-directed action. The brain’s representation of the body in space is thought to be generated through a process of multisensory integration of visual, tactile and proprioceptive signals. In this study, we devised a setup that allowed us to displace participants’ right hand without their subjective awareness. We accomplished this task by instructing the participants to view a live video feed of their hand from the first-person perspective. In the active condition, we used a sensorimotor illusion that caused the participants to actively but unknowingly displace their unseen right hand to a location 8 cm lateral to the image of their hand. In the passive condition, we mechanically displaced the participants’ hand—at a slow, unnoticeable velocity—to the same location. We found that during active displacement, the participants indicated that the location of their hand was closer to the digital image of the hand rather than the veridical location of the hand, as compared with the passive condition, in which the participants indicated that the locations of their hand were closer to the actual location. These results indicated that, compared with passive displacement, active movements cause greater recalibration of the hand’s spatial position and that the boosted spatial recalibration of hand position sense in the active task is driven by error-based sensorimotor corrections. These results have bearing on the perceptual mechanisms of recalibration of perceived limb location.

## Introduction

Precise knowledge of the limbs’ position in space is fundamental for goal-directed action (Jeannerod et al. 1995). The brain’s representation of the body in space is thought to arise through a process of multisensory integration of visual, tactile and proprioceptive signals (Graziano and Botvinick 2002; Makin et al. 2008). With the discovery of the rubber hand illusion (Botvinick and Cohen 1998), manipulation of the perceived location of one’s hand became possible by inducing the illusion of owning a rubber hand. In the rubber hand illusion, a rubber hand is placed in full view in front of a participant, at an anatomically plausible position, while the participants’ real hand is hidden from sight. The rubber hand and the participants’ real hidden hand are then brushed with two small paintbrushes simultaneously and at the corresponding locations. After a stimulation period of approximately 10–15 s, the majority of participants begins to develop the sensation that the rubber hand is his or her own hand (Ehrsson et al. 2004; Lloyd 2007). This sense of ownership is usually coupled with a shift in the perceived location of the participants’ hand, so that when participants are asked to close their eyes and point toward where they perceive their hand to be located, their pointing responses are biased towards the rubber hand. This bias is referred to as ‘proprioceptive drift’ and is considered a recalibration of perceived hand location based on the processing of multisensory signals to realign the visual and proprioceptive representations of the hand (Tsakiris and Haggard 2005; Ehrsson 2012). Interestingly, although proprioceptive drift often correlates with the subjective rubber hand illusion, this correlation is not always observed (Abdulkarim and Ehrsson 2016; Rohde et al. 2011), and hand position sense may drift without accompanying changes in the explicit sense of hand ownership (Holle et al. 2011; Holmes et al. 2006). Furthermore, changes in proprioception have been observed in experiments without any manipulation of ownership, in which the perceived location of one’s limb in space was observed to drift due to experimentally induced visuo-proprioceptive mismatches (Brown et al. 2003; Patterson et al. 2017; Beers et al. 1996; Robert et al. 2002). These findings demonstrate that substantial recalibration of position sense can occur in the absence of consciously perceived changes in ownership of the body.

In a previous experiment conducted in our lab (Abdulkarim and Ehrsson 2016), we have examined the relationship between changes in hand position sense and the subjective feeling of ownership in the rubber hand illusion. We devised a motor-controlled mechanical setup that allowed us to displace the participants’ hidden right hand medially or laterally so slowly that the participants did not notice the displacement. We then elicited the rubber hand illusion with synchronous seen and felt brushstrokes, while the participants’ hidden right hand was slowly displaced toward or away from the rubber hand. We quantified the subjective illusion using a standard questionnaire and measured the change in perceived limb location using the ‘proprioceptive drift’ pointing task. Our results showed that proprioceptive drift could be dissociated from the subjective illusion. The slow mechanical manipulation of hand position sense toward or away from the rubber hand had no effect on the subjective illusion. However, we also found that the participants were surprisingly accurate in localizing the hand’s new position after the mechanical displacement. We found this result surprising because the participants had been unaware of the displacement and cognitively thought that the hand was still in its original starting position. However, when asked to close their eyes and point toward their right hand with their left index fingers, the participants were quite accurate in localizing the hand’s new position. Even when the participants were unaware of the displacement, the participants were still able to indicate the location of the unseen hand only 40% short of the full distance in the right direction.

A similar recalibration of hand position sense that appears to occur without conscious awareness has been described in an interesting study by Newport and colleagues (Newport and Gilpin 2011). These authors have used a sensorimotor illusion in which the participants actively but unknowingly displaced their hand but perceived their hand to be in the original starting position. In this illusion, the participants place their hand inside a box with a screen mounted on top of it. Through a reflection of the screen, the participants view—from the first-person perspective—a live video stream of their hand inside the box in the same horizontal plane as their real hand; thus as if they were looking down at their own hand placed inside the box. The video stream of their hand is then manipulated and slowly shifted medially, without the participants’ awareness. The participants then unknowingly make correctional movements laterally to maintain their hand at the same visual location. At the end of the experiment, when their hand have been displaced and their hands now are 12.5 cm farther apart, the image of their right hand is occluded, and participants are asked to reach over and grasp their right hand with their left hand. However, when they reach over, they reach toward a portion of empty space and are amazed that their hand has ‘disappeared’. In this experiment, the participants cognitively think that the hand has remained at the original location, when the hand has in fact changed position through an unconscious active recalibration process. In a follow-up study, Bellan and colleagues examined the relationship between the visual and proprioceptive contributions to the disappearing hand trick in detail (Bellan et al. 2015). In this study it was shown that the participants initially after the introduction of the visuo-proprioceptive mismatch relied most on the visual trace of the hand, but that with time (as the visual memory of the hand faded), they relied more on proprioception and thus made more correct judgments of their veridical hand position.

These two different experiments demonstrate that the representation of the limbs in space can undergo recalibration in the absence of awareness. However, there is an interesting difference between our experimental results and those of Newport & Gilpin. Both setups involve displacement of the participants’ real hand without their awareness, but in our setup, the displacement is induced passively by slowly moving the hand with a mechanical device and in Newport and Gilpin’s study, the displacement is performed actively by the participants themselves by an unconscious active visuomotor recalibration process. Interestingly, the results appear to go in opposite directions. In our study (Abdulkarim and Ehrsson 2016), the participants were relatively accurate in localizing their hand’s new veridical position, whereas in ‘the disappearing hand trick’, the participants localize their hand more towards the illusory location of the digital image of the hand rather than the actual displaced hand’s location.

In the present study, we set out to investigate this difference between our previous results and the results from Newport et al. We hypothesize that a critical factor in multisensory recalibration of hand position is whether the process involves self-generated active motor commands. More specifically, we expected that a passive hand condition would involve only a spatial recalibration process based on visuo-proprioceptive integration and that an active hand condition would additionally engage a visuomotor process based on error-feedback. Thus, we expected greater illusory shifts in the hand position sense in the active condition compared with that in the passive condition and conversely more accurate localization of veridical hand position in the passive condition. We also obtained questionnaire data to verify that the participants were unaware of the experimental manipulations of hand position.

## Materials and methods

### Ethics statement

All participants provided written informed consent prior to their participation. All experiments were approved by the Regional Ethical Review Board of Stockholm and conformed to the declaration of Helsinki.

### Participants

A total of 13 healthy adults were recruited with the following distributions: eight females and five males; mean age 28 ± 10 years. The participants were recruited primarily from the student population in Stockholm. The participants received a cinema ticket as compensation for their participation in the experiment.

### Setup

#### The hand illusion box

**Fig. 1:**
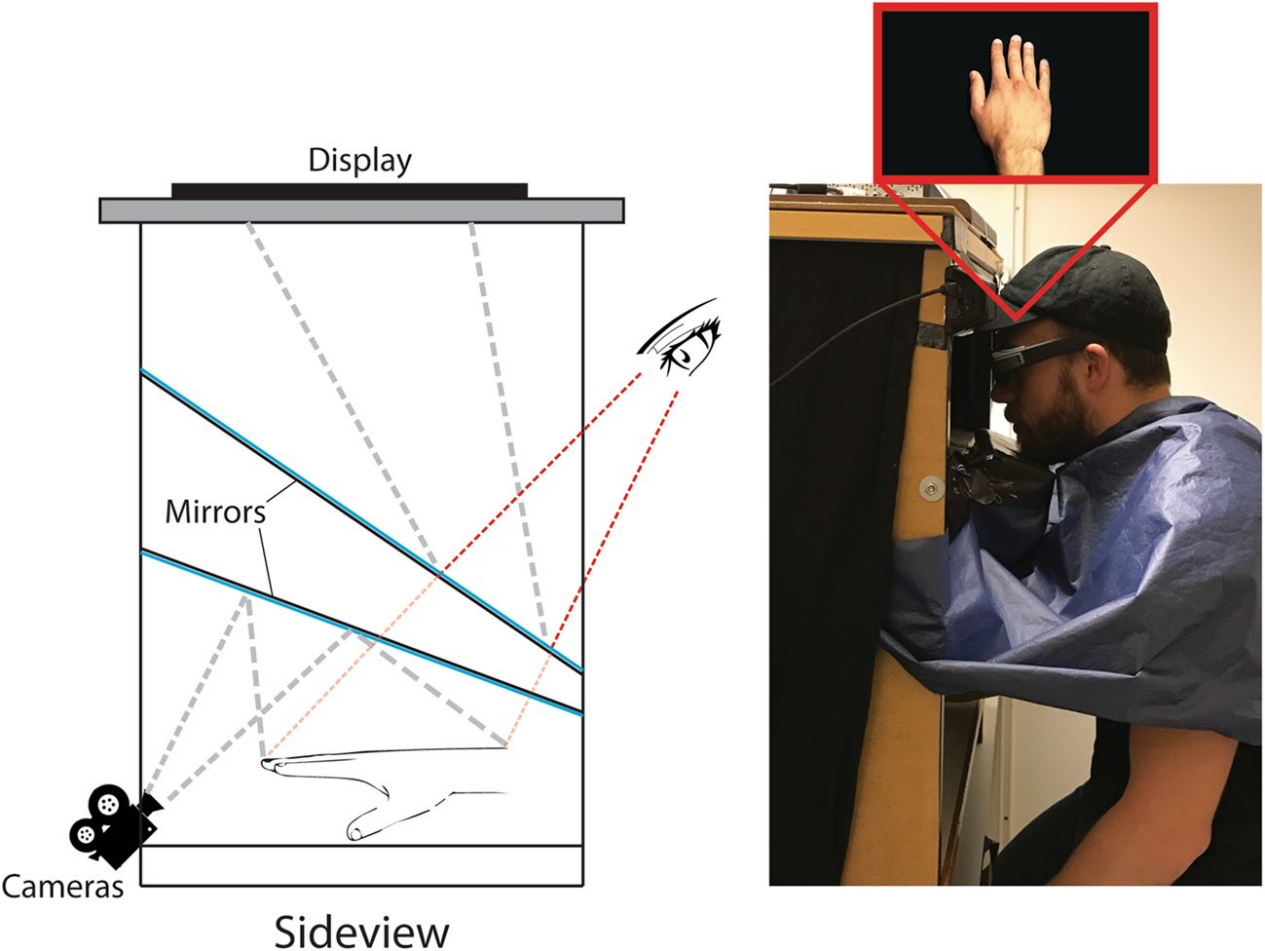
Schematic illustration of the hand illusion box setup on the left and photo of the constellation with a participant on the right. The box consists of a wooden frame, two mirrors, two cameras, a 3D display and a pair of 3D glasses. The image of the hand is reflected in the mirrors, recorded by the cameras and then relayed to the display. The display is placed on top of the box facing downward; the image from the display is thus reflected in a second mirror before reaching the eyes of the participants. This setup results in the participants seeing their hand from the first-person perspective when they look down at the mirror. The size of the viewed hand inside the box was 90% of the actual hand size

We used ‘The hand illusion box’ setup previously developed in our laboratory. The setup draws inspiration from the ‘MIRAGE box’ setup by (Newport and Gilpin 2011). The hand illusion box consists of a wooden frame that measures 76 × 77.5 × 51 (W × H × D) cm and contains a series of mirrors, two cameras (AVT ‘Guppy Pro’, Stadtroda, Germany), a 3D compatible computer screen (ASUS VG278HE, Taipei, Republic of China) and 3D glasses and sensor (NVIDIA 3D Vision, California, USA) (Fig. 1). The cameras were connected to a computer that was further connected to the display. The cameras thus provided live video images that were relayed to the screen with a delay of approximately 46 ms. The live video from the cameras was filmed in 60 frames per second and the refresh rate of the display was 120 Hz (60 Hz for each camera). This setup allowed us to display a 3D video of the participants’ right hand to them from the first-person perspective when they are seated in front of the hand illusion box, and their hand is placed inside the box. The digital images of the hand were precisely displayed on the screen to match the angle of the person looking down at his or her own hand and the viewed hand was approximately the same size as the real hand (10% smaller). Further, because the videos were relayed through a computer, the videos displayed on the screen could be digitally manipulated in real time.

#### Mechanical hand displacement apparatus

**Fig. 2:**
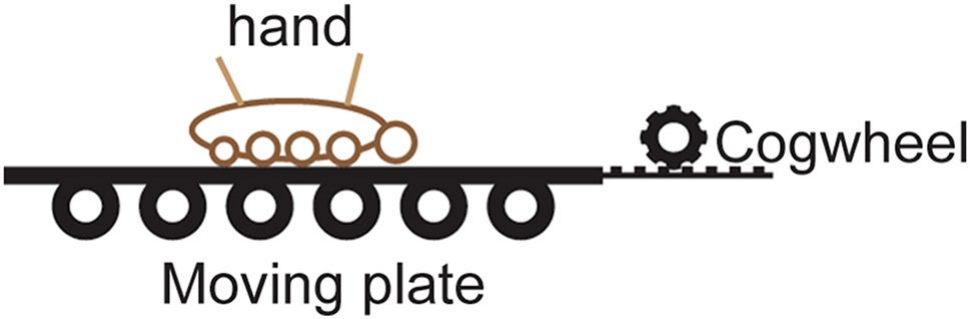
Illustration of the mechanical hand displacement apparatus

An apparatus designed to displace the participants’ right hand was devised. The apparatus consisted of two sheets of Plexiglas glued together and separated by Styrofoam and a metal plate. The lower sheet rested on a set of plastic cylinders that in turn rested on a rubber mouse pad (Fig. 2). The lower Plexiglas sheet was connected to an electrical engine (Micro Motors E192.24.625, Verderio Inferiore, Italy) via a cogwheel and a rack bar. This setup allowed the engine to displace the Plexiglas sheets both laterally and medially with a velocity of 0.9 mm per second. On the basis of previous studies on the sensitivity of limb kinesthesia, adults do not perceive passive angular joint displacements slower than 0.3°/s (Bairstow and Laszlo 1981; Pickett and Konczak 2009). Therefore, we set the speed of the extension or flexion in the elbow joint in the passive displacement condition to 0.3°/s or less. For a forearm of the length 30 cm, this would be equivalent to moving the hand 1.6 mm/s. The setup used in this experiment was constructed by placing the mechanical hand displacement apparatus inside the hand illusion box (see above). The Plexiglas sheets were covered in black cloth to match the background in the box, thus allowing us to manipulate the location of the participants’ hand inside the box passively by instructing the participants to rest their hand on the displacement apparatus or actively by holding the hand above the support surface (akin to Newport’s ‘disappearing hand trick’) (Newport and Gilpin 2011).

#### Digital manipulation

**Fig. 3:**
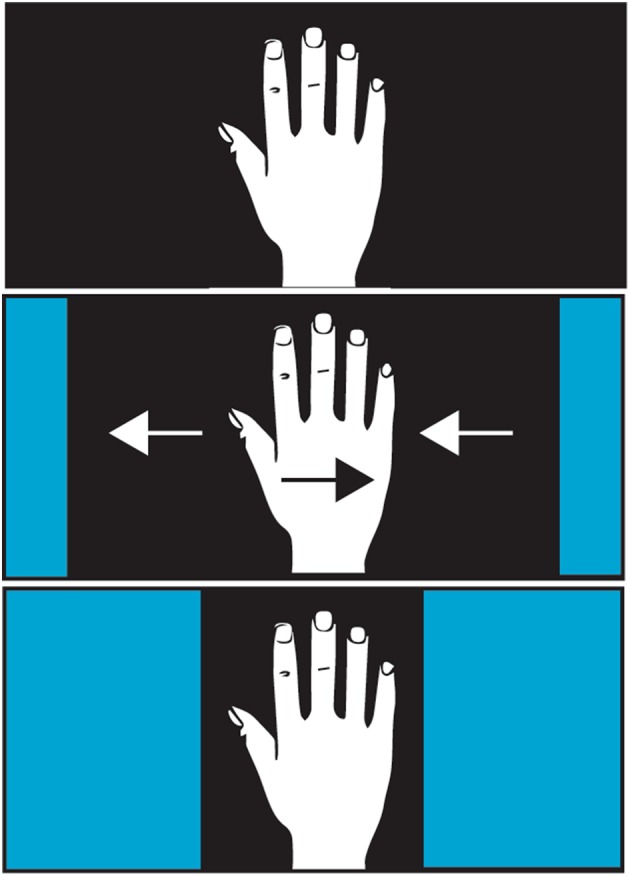
Illustration of the digital manipulation. The participants viewed their hands when the blue margins started to contract inward-like curtains. Simultaneously, the video stream of the hand started to shift leftwards, and this leftward shift was counteracted by either the participants themselves (active trials) or by the mechanical hand displacement apparatus (passive trials)

Using the setup with the hand illusion box and the mechanical hand displacement apparatus, we digitally manipulated the videos of the participants’ hand that they viewed when they were seated in front of the hand illusion box and placed their right hand inside the box. The manipulation consisted of digitally cropping the video of the hand from both the right and left margin of the visual field akin to curtains closing in from the left and right side. Specifically, the cropping consisted of two blue fields move in from the sides of the screen towards the center, similar to the manipulation in the disappearing hand trick (Newport and Gilpin 2011). The blue fields stopped when the distance between the blue fields (i.e., the area of the screen showing the hand) was 12.5 cm wide. Cropping the left and the right margin while simultaneously shifting the live video stream to the left caused the visual image of the hand to be displaced to left. Thus, to keep the hand in middle of the visual field as instructed, the participants very slowly and unknowingly displaced their hand rightward, away from the body midline. This video image manipulation was conducted so slowly that the participants did not notice the drift of their hand image, yet it triggered very small correctional movements of the hand without the participants consciously noticing these movements. In the passive trials, participants simply rested their hand on the moving hand displacement apparatus, and this apparatus slowly moved their hand laterally to correct for the digital shift of the video stream. An electrical engine powering a cogwheel that was connected by a rack bar to the moving plate (on which the hand rested) generated the movement of the hand displacing apparatus. To the participants, their hand appeared to be in the same position, in the middle of the screen during the entire trial, when in fact the participants were either making small correctional movements to compensate for the digital shifts that were imposed on the video streams, or the mechanical device was displacing their hand. In the two no-movement control conditions, the left and right margins were cropped without shifting the live video stream, thus keeping the participants’ hand centered in their original position (Fig. 3). During the active no-movement condition, the participants thus simply held their hand just above the support surface, and in the passive no-movement condition, the hand rested on the immobile platform without any movements being generated by the apparatus.

### Study design and aim

The aim of this experiment was to directly compare active vs passive unaware displacements of the right hand with respect to the perceived location of this hand. On the basis of our previous data (Abdulkarim and Ehrsson 2016) compared with the study by Newport et al. (2011), we hypothesized that the participants would be more accurate in localizing the veridical position of their right hand when it had been displaced passively rather than actively.

The experiment used a 2 × 2 factorial design (active vs passive × displaced vs static). The crucial manipulation was active or passive displacement of the participants’ hand or kept immobile through actively holding the hand in the air or relaxing it on a support surface (as described in detail above). In the active conditions, the participants were instructed to hold their hand still in the air. In the active displaced condition, the digital shift of the live video image was corrected for by the participants who unknowingly made the opposite movement with their hand. In the active static condition, which served as a control condition, no digital image shift occurred, and the subjects simply held their hand in a static position. In the passive conditions, the participants rested their hand on the upper Plexiglas sheet of the mechanical hand displacement apparatus. In the passive displaced condition, small hand movements were made to correct for the digital manipulation by the mechanical hand displacement apparatus that displaced the participants’ hand without the subjects’ awareness. In the passive static conditions, no digital shift was imposed on the image of the hand, and the subjects rested their hand on the upper Plexiglas sheet of the mechanical hand displacement apparatus, which was kept static. The participants’ right hand was displaced 8 cm laterally in each active or passive displaced trial, which lasted for 90 s. In the static control conditions, the video images of the participants’ hand were not shifted, and the mechanical hand displacement apparatus was not switched on, thus causing the participants’ real hand to remain at the same position throughout the trials.

### Outcome measures

#### Hand localization task

The recalibration of hand position sense was measured by instructing the participants to manually indicate the perceived location of their right hand with their left hand. The procedure consisted of the following four steps. First, immediately following the end of one trial, the participants were instructed to close their eyes and raise their left hand (which was resting on their lap), while their right hand remained inside the hand illusion box. The experimenter then placed the participants’ left index fingers on a rod (with randomized starting positions) that acted like a metal ruler placed above the participants’ right hand inside the hand illusion box. Immediately after this step, the participants had to slide their left index fingers along the rod until they felt that their left index fingers were precisely above the tips of their right index fingers, at which point they would stop and utter the Swedish word for ‘here’. The experimenter noted this location and moved the participants’ left hand back to their original relaxed position, and the participants were instructed to open their eyes and again look at the screen. The hand position sense recalibration was calculated by subtracting the reported position of the right index finger before the trial started from the reported location of the right index finger at the end of the trial. The hand localization task was repeated three times.

#### Questionnaire

At the end of the experiment, the participants filled out two questionnaires. The first questionnaire assessed whether they felt ownership of their hand, as viewed through the 3D display. We expected them to report ownership because the observed location of the hand matched the perceived location of the hand, and this spatial congruency between vision and proprioception should facilitate a feeling of hand ownership (Gentile et al. 2013). This questionnaire consisted of three statements that the participants rated on a seven-point Likert scale that ranged from − 3 to + 3 (Table 1), where + 3 meant “fully agree”, − 3 meant “fully disagree”, and 0 “I neither agree or disagree”. The first statement (S1) was aimed at capturing the feeling of ownership of the hand, whereas the other statements (S2, S3) were used as controls for unspecific task compliance and suggestibility effects.

**Table 1:** The statements used in the first questionnaire

S1	It felt as if the hand I saw belonged to me
S2	It felt as if I was looking at somebody else’s hand
S3	I could no longer feel my hand

The second questionnaire served to probe whether the participants had sensed the displacement of their hand during the experiment, and whether they had determined the experimental manipulation in another way. This questionnaire was handed out at the very end of the experiment. The questionnaire consisted of five questions (Q1–Q5, Table 2), and all questions, with the exception of one (Q3), were open ended. Q3 was a forced choice question (yes/no).

**Table 2:** The second questionnaire that was handed out at the very end of the experiment

Q1	What do you think the aim of the experiment was?
Q2	In what way do you think the trails differed?
Q3	Did you at any point during the experiment feel that your real hand was moving? (Y/N) forced choice
Q4	If ‘yes’ on Q3, please describe the movement
Q5	Do you have any other comments regarding the experiment?

Question 3 (Q3) was a forced choice question in which the participants had to choose between ‘yes’ or ‘no’. The original questionnaire was given in Swedish

### Procedure

The participants were seated next to the hand illusion box and provided oral and written information about the experiment. Informed consent was collected from the participants. The experimenter performed a demonstration of how the hand localization task would be conducted. The participants were then seated in front of the hand illusion box and wore earplugs, headphones, and 3D glasses (NVIDIA). They were then instructed to place their right hand in the hand illusion box, in the middle of the image (i.e., on top of the moving apparatus), which corresponded to a position straight in front of their bodies (in the body midline). The screen was then blanked (completely blue), and the participants were instructed to close their eyes and perform the hand localization task. The trial was then initiated, and the participants were instructed to keep their hand in the middle of the screen in the active trials or to just relax and look at the screen in the passive trials. At the end of the active trials, the participants were required to rest their hand on the moveable plate; they were instructed to simply ‘put their hand down’. This ensured that the hand localization task would be well matched between the active and the passive conditions. Each trial was 90 s long, and in that time, the participants’ hand was displaced 8 cm laterally in the displaced conditions, either actively or passively, whereas they remained in the starting position in the static conditions. The conditions were counterbalanced within and across participants, to eliminate order effects. Each condition was repeated three times for the hand localization task.

### Statistical analysis

The number of participants recruited was based on previous studies of the rubber hand illusion that had used similar outcome measures (Botvinick and Cohen 1998; Guterstam et al. 2011; Rohde et al. 2011; Tsakiris and Haggard 2005) as well as some of the literature on visuo-proprioceptive adaptation (Bellan et al. 2015, 2017; Newport and Gilpin 2011). All statistics were calculated using SPSS for Windows, release 22.0 (IBM Corp., Armonk, NY). The hand localization data from each participant were pooled into one dataset per condition. Each dataset was tested for normality using one-sample Kolmogorov–Smirnov test. If a dataset deviated significantly from normality, analyses were conducted using non-parametric tests, e.g., Wilcoxon signed-rank test. To test for interaction effects in a 2 × 2 factorial design with non-normally distributed datasets, we calculated the numeric difference between the two levels of each factor and compared them using Wilcoxon signed-rank test. If the datasets were normally distributed, we used repeated measures analysis of variance (ANOVA) to test for main effects and interactions, followed by paired *t* tests for planned comparisons in line with our a priori hypotheses. The effect sizes for ANOVAs were calculated using *η* = SSeffect/SStotal, (SS = Sum of Squares), and the effect sizes for the planned comparisons were calculated using Cohen’s d, according to Eq. 8 from (Morris and DeShon 2002). The effect sizes for the non-parametric comparisons were calculated as *r* = *Z*/√(*N*) (*N* = total number of observations), as described in (Rosenthal 1994). The questionnaire data were considered to be ordinal scale data and thus analyzed using Wilcoxon signed-rank test.

## Results

### Hand localization

**Fig. 4:**
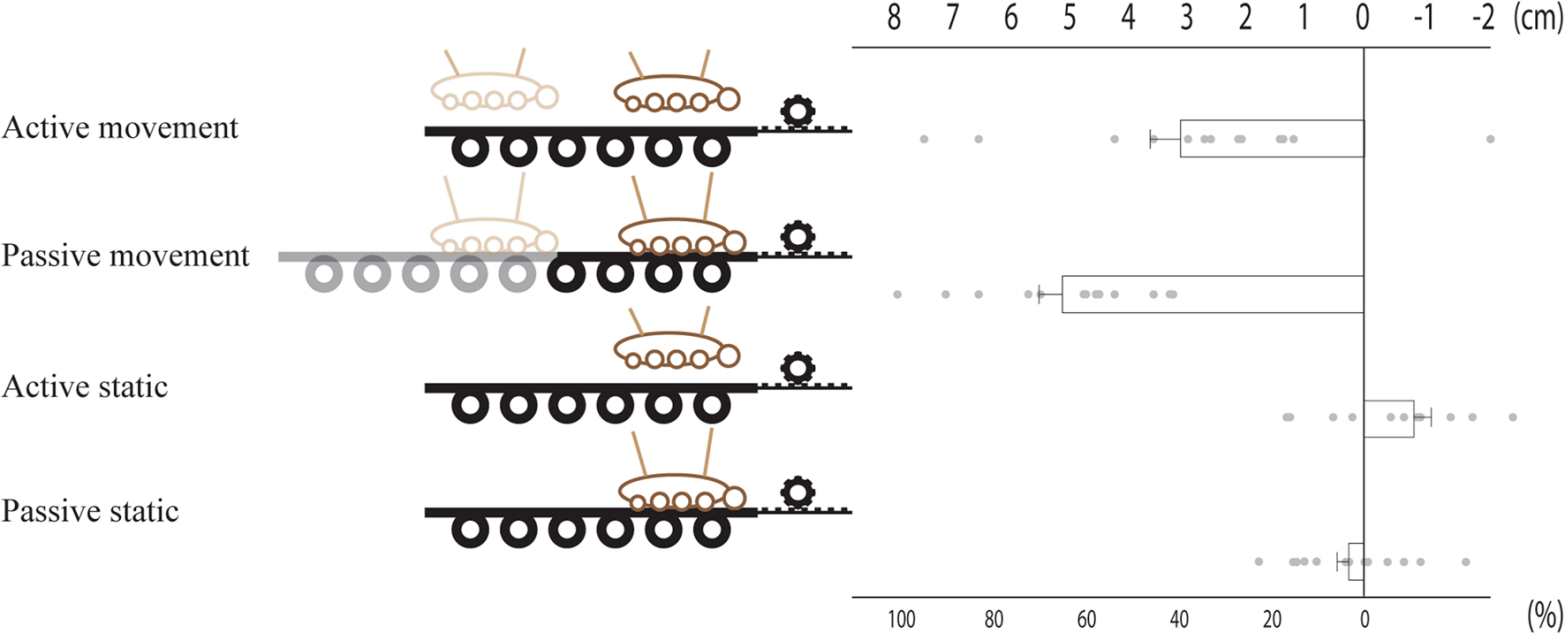
The results from the hand localization task. The hand was displaced from position ‘0’ to ‘8’ in the displacing trials, thus indicating the lateral displacement in cm. The means and standard error of the mean for each condition were as follows: ‘active movement’ (3.1 ± 0.7), ‘passive movement’ (5.2 ± 0.4), ‘active static’ (− 0.7 ± 0.3) and finally ‘passive static’ (0.2 ± 0.2). The bars indicate the mean difference between the judgment of hand localization before and after the displacement. Error bars indicate standard error of the mean. ** indicates *p* < 0.01

The hand localization task revealed that the participants were more accurate (i.e., closer to the hand’s veridical position) when judging the location of their right hand after passive displacement rather than active displacement (Fig. 4). A main effect of type of displacement (active vs passive) *F*(1,12) = 23.482, *p* < 0.001, *η* ^2^ = 0.2515 and a main effect of movement (displaced vs static) *F*(1,12) = 158.942, *p* < 0.001, *η* ^2^ = 0.704 were shown by 2 × 2 ANOVA. Furthermore, a significant interaction was also observed (type of displacement × movement) *F*(1,12) = 8.227, *p* = 0.014. We further used paired *t* tests to compare the specific conditions, and these comparison tests revealed a significant difference between the active movement and the passive movement conditions *t*(12) = 4.630, *p* = 0.001, *d*  = 1.220 (two tailed). These results indicated a significant difference in hand localization that depends on whether the limb has been displaced actively or passively, results in line with our a priori hypothesis.

### Questionnaire

**Fig. 5:**
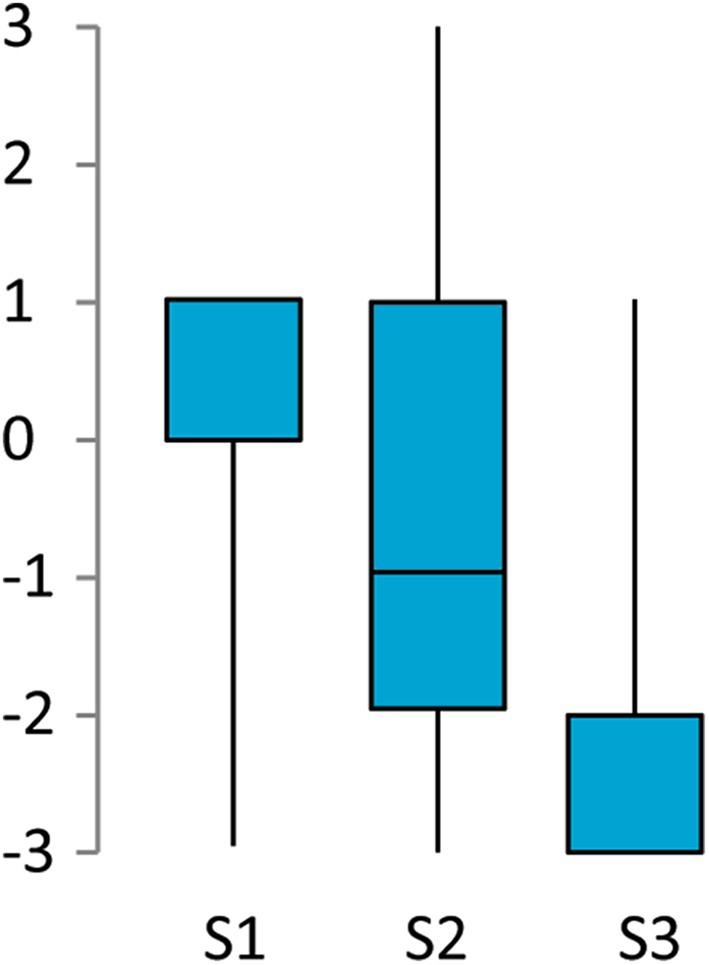
The results from the first questionnaire are presented as a boxplot. The questionnaire was administered at the very end of the experiment and pertained not to any specific condition but instead to the overall experience. Statement 1 refers to ownership over the viewed hand, whereas Statements 2 and 3 were used to control for task compliance and suggestibility. The statements were rated on a 7-point Likert scale ranging from − 3 (completely disagree) to + 3 (completely agree). The median for statement 1 was (1), the median for statement 2 was (− 1) and the median for statement 3 was (− 3)

The results from the first questionnaire showed that the participants’ ratings of the illusion statement were higher than the ratings of the control statements (Fig. 5). The statistical analysis revealed the data to be non-normally distributed; therefore, Wilcoxon signed-rank test was performed. Wilcoxon test revealed a significant difference between the ratings for the illusion statement and the ratings for the pooled control statements, *Z* = 2.989, *p* = 0.003, *r* = 0.229. These results suggested that the participants perceived the viewed hand inside the hand illusion box as their own.

**Fig. 6:**
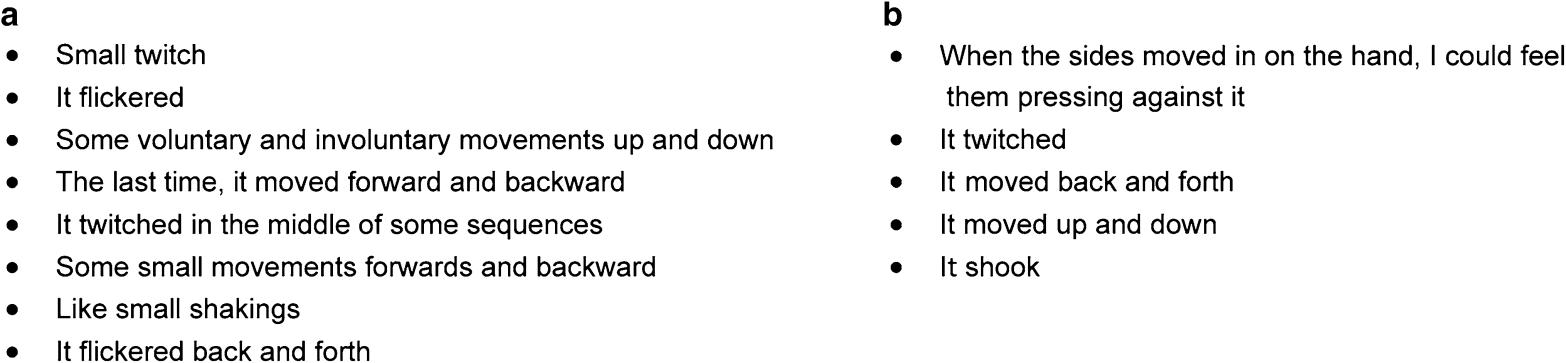
The responses to Q4 of the second questionnaire (Table 2). The responses were given in Swedish and have been translated into English

The second questionnaire showed that five participants reported ‘no’ on the forced choice question (Q3), whereas eight reported ‘yes’. However, when asked to freely describe what they perceived in terms of hand movements, none of the eight participants reported a movement that could be attributed to our manipulation, thus indicating probable task compliance and suggestibility. The responses of the participants to Q4 are translated and reported in Fig. 6a.

## Discussion

We investigated differences in hand localization when the hand was displaced actively or passively during a multisensory illusion in the absence of awareness of movement. The main finding was that the participants were significantly more accurate in localizing their hand’ veridical locations when the hand was displaced passively rather than actively. These results suggested that different central processes are involved in active and passive multisensory recalibrations of hand position sense and that these processes can operate without subjective awareness. These findings support the hypothesis that recalibration of hand position sense during active movements involves an error-based sensorimotor mechanism involving efference copy, which is not engaged in passive conditions where visuo-proprioceptive integration is sufficient. This conclusion is important because it suggest that previous findings of hand recalibration in the “disappearing hand trick” (Newport and Gilpin 2011; Bellan et al. 2015, 2017) could be driven by such error-based sensorimotor process engaged in the active tasks used in these studies. More generally, our results are important because they suggest that the active maintenance of stable limb postures in space involves sensorimotor processes and multisensory interactions that we are not aware of.

In a multisensory integration framework, the Maximum Likelihood Estimation (MLE) (Ernst and Banks 2002) states that when integrating sensory information from multiple senses to form a coherent percept, the sense with the highest reliability (i.e., lowest variability) will be weighted the highest. With regards to our setup, this means that the active place holding of the participant’s hand in active conditions decreases proprioceptive acuity (due to muscle fatigue) (Miura et al. 2004; Voigt 1996), which would thereby decrease the reliability of proprioception. Thus, more weight would be added to the visual position of the hand when judging the location of one’s hand. This relates to studies by Bellan et al., which employ a MLE approach to explain the disappearing hand trick (Bellan et al. 2015, 2017). In these studies, the authors showed that participants initially localize their hand closer to the visual hand, but as the visually encoded position of the hand decays, they shift to rely more on proprioception and localize their hand to its veridical location. However, it is worth noting that Bellan et al. only tested active condition, and did not compare active and passive conditions. Furthermore, they used a perceptual localization task that does not require any pointing with the contralateral hand, so it remains unclear whether these results can translate between our setup and their setup. In addition, other studies on unconscious sensorimotor adaptation make use of a setup in which sensory feedback from volitional action is manipulated to induce motor adaptation. Interestingly, when these studies compared active and passive movements, the difference was not very large, in contrast to our experiments (Hart et al. 2016; Ruttle et al. 2016; Zbib et al. 2016). However, this can be explained by the fact that in these studies, the movements in both active and passive conditions were consciously perceived by the participants and were not intended to be unconscious. Thus, the lack of differences could be due to more salient error signals in both the active and passive conditions.

Our finding of greater recalibration of hand position sense in the active condition compared with the passive condition is perhaps more similar to classical findings on sensorimotor recalibration in prism adaption. In these studies, active goal-directed actions have been found to give rise to a faster recalibration of the motor system than occurs when the same movements are performed passively by the experimenter (Beckett 1980; Fernández-Ruiz et al. 2004; Held and Hein 1958; Mikaelian and Held 1964; Welch et al. 1979). This result has been used as evidence favoring a model of motor recalibration that requires self-generated motor output to update the proprioceptive and visual system (Beckett 1980; Mikaelian and Held 1964). Further, prism adaptation aftereffects have been shown to decay faster during active movement conditions compared with passive conditions (Fernández-Ruiz et al. 2004). Our results are in agreement with the previous prism adaptation literature in that our active hand displacement condition, as compared with the passive condition, was associated with greater recalibration in hand position sense. This finding suggests that error signals, which arise when the internal copies of efferent motor commands are compared with afferent sensory feedback, may contribute to the visuo-proprioceptive recalibration of hand location in the present paradigm as well as in prism adaptation.

However, there are several differences between our paradigm and the typical prism adaptation experiments that make direct comparisons of the results both difficult and interesting. First, an important difference between the recalibration of hand position in the hand illusion box setup and prism adaptation is that the present changes in hand position were unnoticed by the participants. In prism adaptation studies, participants are typically instructed to perform a reaching movement toward a target, and then they grossly miss during the initial phases of the adaption period until visual, proprioceptive and motor representations have been realigned. These large errors in pointing responses are also seen during the prism adaptation aftereffect when the prisms are removed. In both phases, these errors are very clearly perceived by the participants at the level of conscious awareness. In contrast, in the present active conditions, the participants were unaware of the very slow and gradual movement of their hand. The participants were aware that they were holding their arms extended in the box, but the small corrective movements were not consciously noticed. If we assume that the same mechanism involving comparisons of efferent motor commands and sensory feedback is utilized for sensorimotor recalibration in prism adaption and the present hand illusion box paradigm, this assumption would suggest that the actual recalibration process does not depend on perceptual awareness. In comparing the prism adaptation literature to our results, one might even speculate that conscious intention of the self-generated movement is not necessary for visuomotor adaptation, but in our active conditions, the participants still had the conscious intention to hold their hand still in the air, which requires an active effort, so we cannot exclude the possibility that conscious intention played a role; however, this possibility should be further investigated. Second, the type of error signal is very different between the two paradigms. In the present active condition, the participants received very weak error signals that their motor commands directed to maintaining the arm in a static position in mid-air is slightly off and therefore require adjustment. As described, these error corrections are so small that participants are not aware of them. In contrast, during prism adaption, there is a very strong error signal when the hand misses the target. Of course, we cannot exclude the possibility that the sensorimotor comparator mechanisms are different for goal-directed action with large and explicit errors between the goal and the outcome and the postural control with subtle errors between the intended and actual limb posture in our paradigm. In sum, given the considerations above, there are interesting parallels between the recalibration of hand position in our paradigm and visuomotor adaption in the prism adaptation literature that indicate specific roles for active motor commands. Thus, error-based corrections based on active motor commands may not only play a role in voluntary goal-directed movements as occurring in the prism adaptation paradigms but also in the active maintenance of stable postures without any consciously perceivable errors as in the present experiment.

A major assumption of the present study was that the participants were unaware of the displacement of the hand in the active and passive conditions. However, in general terms, it is difficult to prove that participants are completely unaware of a process. First, we cannot rule out that the participants did not have any low-level awareness at some points during the experiment and were unable to report this awareness. Perhaps they forgot or deemed the awareness so weak so that it was below their own “criterion” for reporting it. Second, the strict psychophysical definition of an unaware process is that participants cannot discriminate it above chance level accuracy (Peters and Lau 2015; Sand and Nilsson 2016), but our experiments were not designed as a two-forced-choice paradigm to test chance level accuracy. However, on the basis of our questionnaire data, we are still fairly confident that participants were not subjectively aware of the hand displacements throughout the experiments. In our post-experimental questionnaires, we did ask the participants to make a forced choice (Q3 of the second questionnaire, see Table 2) with regard to whether they, at any point during the experiment, sensed that their hand had moved. Importantly, the participants either denied feeling this sensation, or in cases in which they gave an affirmative response, they subsequently described a movement that could not possibly be related to the experimental manipulation, thus suggesting that they were confabulating (see Fig. 6). Thus, even if we cannot be fully certain that the participants were completely unaware of the hand movements at all times, we believe that the response to the second questionnaire at least demonstrates that putative conscious awareness of the movements under discussion was extremely limited.

Another question is how our results relate to the recalibration of hand position sense seen in limb ownership illusions. In the rubber hand illusion, participants view a rubber hand placed in front of them that is stroked with a small paintbrush, while their hidden hand is synchronously stroked with a similar paintbrush at the corresponding location. After several seconds of this stroking, the participants start to experience the rubber hand as their own. Interestingly, when participants are asked to close their eyes and point toward the location of their hand, they are biased in the direction of the rubber hand. In this case, the illusory ownership of an inanimate limb causes a recalibration of the proprioceptive position of the participants’ hand. However, similar to the results for the passive conditions in our study, the participants were still surprisingly accurate in localizing their real hand. The bias in the perceived location of their hand toward the rubber hand was usually in the order of magnitude of 10–15% of the total distance between the rubber hand and the participants’ real hand. Thus, without appropriate motor output, the perception of the hand’s position in space appears to be quite robust, even in situations in which there is salient sensory information and a subjective illusion suggesting a change in hand location. However, the conditions in the present study differ from the situation in the rubber hand illusion. In the rubber hand illusion, the visual information suggests that the participants’ hand have a new position, while the proprioceptive information suggests that the hand is in its original position. In contrast, in the passive conditions in our study, the proprioceptive information suggests that the participants’ hand have been displaced, whereas the visual information suggests that the hand is in its original position. Importantly, in both the active and passive conditions of the present study there were changes in position sense of the hand occurring without accompanying explicit changes in perceived ownership of the hand. The questionnaire data suggested that participants maintained a subjective sense of ownership of the hand observed in the hand illusion box. Thus, the present study provides further evidence that position sense and subjective ownership of a hand in view can deviate from each other and underscores the importance of adopting caution when interpreting changes in position sense (proprioceptive drift) as objective evidence of limb ownership (Abdulkarim and Ehrsson 2016).

In summary, we investigated the differences in hand localization after unconscious active and passive displacement of participants’ right hand in a within-subjects design. Our results showed that participants were significantly more accurate in localizing their hand’ new veridical location when the hand had been displaced passively rather than actively in a multisensory recalibration paradigm. Thus, active movements cause greater unconscious multisensory recalibration of hand position sense than passive movements, supporting a model where efference copy-based error signals contribute to the recalibration process when active postural corrections are engaged.
